# Genomic selection in pig breeding: comparative analysis of machine learning algorithms

**DOI:** 10.1186/s12711-025-00957-3

**Published:** 2025-03-10

**Authors:** Ruilin Su, Jingbo Lv, Yahui Xue, Sheng Jiang, Lei Zhou, Li Jiang, Junyan Tan, Zhencai Shen, Ping Zhong, Jianfeng Liu

**Affiliations:** 1 College of Science, China Agricultural University, Beijing, 100083 China; 2 College of Animal Science and Technology, China Agricultural University, Beijing, 100193 China; 3 Mathematics and Interdisciplinary Science Research Center, China Agricultural University, Beijing, China

## Abstract

**Background:**

The effectiveness of genomic prediction (GP) significantly influences breeding progress, and employing SNP markers to predict phenotypic values is a pivotal aspect of pig breeding. Machine learning (ML) methods are usually used to predict phenotypic values since their advantages in processing high dimensional data. While, the existing researches have not indicated which ML methods are suitable for most pig genomic prediction. Therefore, it is necessary to select appropriate methods from a large number of ML methods as long as genomic prediction is performed. This paper compared the performance of popular ML methods in predicting pig phenotypes and then found out suitable methods for most traits.

**Results:**

In this paper, five commonly used datasets from other literatures were utilized to compare the performance of different ML methods. The experimental results demonstrate that Stacking performs best on the PIC dataset where the trait information is hidden, and the performs of kernel ridge regression with rbf kernel (KRR-rbf) closely follows. Support vector regression (SVR) performs best in predicting reproductive traits, followed by genomic best linear unbiased prediction (GBLUP). GBLUP achieves the best performance on growth traits, with SVR as the second best.

**Conclusions:**

GBLUP achieves good performance for GP problems. Similarly, the Stacking, SVR, and KRR-RBF methods also achieve high prediction accuracy. Moreover, LR statistical analysis shows that Stacking, SVR and KRR are stable. When applying ML methods for phenotypic values prediction in pigs, we recommend these three approaches.

## Background

In 2001, Meuwissen et al. [1] proposed a new molecular breeding technique, namely genomic selection (GS). GS is a genetic evaluation strategy that utilizes high-density single nucleotide polymorphism (SNP) markers across the entire genome to estimate genomic estimated breeding values (GEBVs) of individuals [2]. Compared with traditional breeding values (BV) estimation methods that rely on pedigree information, GS can better account for the genetic variation among generations, improve the accuracy of breeding value prediction, shorten generation intervals, and accelerate genetic progress. Furthermore, GS has shown promising prediction results in the traits with low heritability [3].

There are two kinds of classical GS methods: one is the method based on best linear unbiased prediction (BLUP), the others are Bayesian methods. There are many methods based on BLUP, which integrate more information and construct new matrices to replace the pedigree relationship matrix (**A**) in BLUP. Taking Genomic BLUP (GBLUP) proposed by VanRaden [4] as an example, it replaced the pedigree relationship matrix (**A**) in BLUP with a genomic relationship matrix (**G**) to estimate GEBVs. GBLUP has higher accuracy compared to BLUP. Bayesian methods was firstly proposed by Meuwissen [2] in 2001, then a lot of Bayesian linear regression methods were proposed, such as BayesC, BayesCπ, and BayesDπ [5]. Although Bayesian methods have better accuracy, their time-consuming nature makes it challenging to apply them in actual production. Furthermore, GBLUP and Bayesian methods estimate GEBVs using linear models that consider only additive effects, while ignoring interaction effects and epistatic effects [6]. This may negatively impact the accuracy of genomic prediction. Furthermore, the requirement for larger storage space escalates as the number of SNP markers increases. Consequently, the efficiency of genomic prediction becomes relatively low. In order to improve the prediction accuracy and computational efficiency, the nonlinear model considering potential interaction information between SNP markers and the methods suitable for high-dimensional data should be developed.

Due to the nonlinearity of ML methods and their advantages in processing high-dimensional data, they have been used for genomic prediction in recent years [7]. Zhao et al. [8] used SVR-rbf, linear SVR, GBLUP and BayesR to estimate GEBVs of eight quantitative for pigs. The results showed that SVR model was a competitive method in genomic prediction. Lee et al. [9] predicted the growth traits by ML methods, including logistic regression, linear SVR, decisions tree and random forest (RFR). They found that RFR performed better than other methods. Wang et al. [10] used SVR, KRR, RFR and Adaboost.R2 to improve the accuracy of GP for reproductive traits in the Yorkshire pigs. They found that Adaboost.R2_KRR consistently performed well. Rocha et al. [11] compared various ML methods to improve the accuracy of pig live weight prediction, Stacking was found to be the best one. Gonzalez-Recio et al. [12] compared the accuracies of Bayesian regressions (BayesA and Bayesian LASSO) and ML methods (Boosting and RFR) for the whole-genome prediction of discrete traits. When a few QTLs regulated traits, ML methods had certain advantages over Bayesian regression. However, when the number of QTLs was large, the difference was very small. Deep learning (DL), as a branch of ML, is an emerging algorithm based on multi-layer neural networks and it is also used in genomic prediction. Abdollahi-Arp Anahi et al. [13] compared the performance of six methods on real and simulated datasets, including the multilayer perceptron network (MLP), convolutional neural network (CNN), RFR, gradient boosting (GB), GBLUP and BayesB). DL algorithms were found to worked well when the sample size was large enough, but their performance was not consistent. Lee et al. [14] introduced a novel genomic prediction algorithm named dengue, which successfully integrated DL networks and GBLUP frameworks. Compared with the GBLUP and Bayesian methods, the experiments showed that deepGBLUP performed well on the cattle dataset. Xiang et al. [15] used four different nonlinear models (SVM, XGBoost, RF, and CNN) on two real pig datasets and compared the results with those using GBLUP, demonstrated that it is feasible and reliable to implement GP with nonlinear models. Liang et al. [16] integrated SVR, KRR and ENET (Elastic net) to constructed a stacking ensemble learning framework (SELF) for genomic prediction. The accuracy of SELF was higher than GBLUP on three real datasets. González-Camacho et al. [17] reported that linear SVR performed the best on the genomic prediction of the wheat rust resistance compared to other ML methods. Cuevas et al. [18] proposed two rbf kernel methods which considered the interaction of genotype and environment (RKHS KA and the RKHS EB). These methods showed consistent increases in GP accuracy compared to GBLUP on two real datasets. Kernel methods are flexible and easy to interpret and have been successfully used in Multi-environment Breeding Trials [19].

The objectives of this paper are (1) comparing the performance of eleven ML methods and GBLUP in predicting the growth and reproductive traits; (2) statistically validating the predictive performance of various algorithms though average rank; (3) using LR statistics to compare the models; (4) pick out ideal ML methods for genomic prediction.

## Materials and methods

In this section, we briefly introduce the datasets and methods used in this paper.

### Dataset

Five distinct pig populations that have been widely applied in prior research were used to evaluate the effectiveness of commonly used ML methods [7, 20, 21]. There were 16 datasets in these five pig populations, covering both growth and reproductive traits. The traits, with heritability ranging from 0.07 to 0.62, were used to ensure the robustness and generalizability of our study.

#### Population 1: PIC population

This population is from a nucleus pig farm, it contains genotype data of 3534 individuals obtained by the Illumina PorcineSNP60 chip, each individual has five distinct traits, but the specific information of the traits is hidden. Each phenotype is either adjusted for environmental factors and rescaled by correcting for the overall mean (traits 3, 4, and 5), or represented a rescaled, weighted average of corrected progeny phenotypes (traits 1 and 2) [22]. In this study, we denote trait 1 to trait 5 as T1 to T5, respectively.

#### Population 2: large white population 1

This population is from a pig breeding farm in Inner Mongolia, China. The growth traits include the age at 100 kg (AGE) and back-fat thickness at 100 kg (BF). In the experiments, these traits are named Z_AGE and Z_BF respectively. The reproductive traits encompass the total number of born piglets (TNB), number of born alive piglets (NBA), and liter weight at birth (LW) [23].

#### Population 3: large white population 2

This population comes from three pig nucleus breeding farms in China. It entails 32,980 SNPs and two growth traits: age at 100 kg (AGE) and backfat thickness at 100 kg (BF). After quality control, there are records of 4146 pigs with the AGE trait, and 4130 pigs with the BF trait [24]. In the experiments, these traits are named Y_AGE and Y_BF, respectively.

For population 2 and 3, the traits AGE and BF were adjusted using the National Swine Performance Recording Standard of China.

#### Population 4: American origin duroc population

This population is sampled from the Wen's Foodstuff Group Co., Ltd. (Guangdong, China), which contains 3770 American origin pigs born between 2013 and 2017, 39,416 informative SNPs and two economic traits, loin muscle area (LMA) and loin muscle depth (LMP) [25]. In the experiments, these traits are named as ALMA and ALMP, respectively.

#### Populations 5: Canadian origin duroc population

This population is also sampled from the Wen’s Foodstuff Group Co., Ltd. (Guangdong, China), which contains 2096 Canadian origin pigs born between 2016 and 2017, 35,850 informative SNPs and two traits: LMA and LMP [25]. In the experiments, these traits are named CLMA and CLMP, respectively.

For population 4 and 5, the traits LMA and LMP were collected by practiced investigators from the 10th-rib to 11th-rib of pigs at the weight of 100 $\pm$ 5 kg, the detailed descriptions of these datasets can be found in [25].

The details of above five populations are in Table 1. To facilitate direct comparisons between different models, the TNB, NBA, and LW traits were analyzed using data exclusively from the first litter size. This approach allows researchers to gain insight into early reproductive performance and compare the predictive power of ML models in this particular context.

**Table 1:** Summary of datasets

Dataset	Trait	h^2^	m	Mean	SD	SNPs
PIC population	T1	0.07	2804	− 0.05	1.21	52,843
	T2	0.16	2715	0.00	1.12	52,843
	T3	0.38	3141	0.71	0.96	52,843
	T4	0.58	3152	− 1.07	2.33	52,843
	T5	0.62	3184	37.99	60.45	52,843
Large white population 1	Z_AGE	0.19	1592	0.00	3.17	35,304
	Z_BF	0.36	1592	0.00	3.13	35,304
	TNB	0.20	1187	0.00	4.06	35,304
	NBA	0.20	1187	0.00	14.60	35,304
	LW	0.23	1187	0.00	2.83	35,304
Large white population 2	Y_AGE	0.23	4146	− 0.07	11.46	32,980
	Y_BF	0.37	4130	− 0.06	2.82	32,980
American origin duroc	ALMA	0.37	3916	42.28	3.78	35,336
	ALMP	0.30	3916	52.36	3.72	35,336
Canadian origin duroc	CLMA	0.38	2127	39.14	4.39	32,909
	CLMP	0.36	2127	47.91	3.78	32,909

h^2^: the heritability of the trait; m: the number of the animal with phenotypes; Mean: the average value of the trait; SD: standard deviation of the trait; SNPs: the number of SNP markers; T1–T5: trait 1- trait 5 of PIC population; Z_AGE and Z_BF: 100 kg age and backfat of Large white population 1; Y_AGE and Y_BF: 100 kg age and backfat of Large white population 2; TNB: the total number of born piglets; NBA: number of born alive piglets; LW: liter weight at birth; ALMA and ALMP: loin muscle area and loin muscle depth of American origin pigs; CLMA and CLMP: loin muscle area and loin muscle depth of Canadian origin pigs

### Methods

Now let us describe our notations. The data set is ${\text{D}} = \{(\mathbf{x}_1, {\text{y}}_1 ), ({\mathbf{x}}_2, {\text{y}}_2 ), \ldots , ({\mathbf{x}}_{\text{m}}, {\text{y}}_{\text{m}} )\}$, where ${\mathbf{x}}_{\text{i}}\in {\text{R}}^{\text{p}}$ is the input, it represents the genotype data of i-th sample, y_i_ is output, it represents the phenotype of i-th sample, i = 1, 2, …, m. ${\mathbf{X}} = \left( {{\mathbf{x}}_{1} ,{\mathbf{x}}_{2} , \cdots ,{\mathbf{x}}_{{\text{m}}} } \right) \in {\text{R}}^{{{\text{p}} \times {\text{m}}}}$, is the genotype matrix whose rows represent the SNP markers, $\bf{\text{y}}={\left({\text{ y}}_{1},{\text{y}}_{2},\cdots ,{\text{y}}_{\text{m}}\right)}^{\text{T}}\in {\text{R}}^{\text{m}}$ is the phenotype vector of m samples. ${\mathbf{I}\in \text{R}}^{\text{m}\times \text{m}}$ indicates the identity matrix. The function ϕ is the map that projects the point **x** to the feature space. **K** is the kernel matrix, ${\text{K}}_{\text{ij}}$ is (i, j) element in **K**.

In this section, we briefly introduce the GS methods used in this paper, including GBLUP, KRR, SVR, ENET, LASSO, random forest regression (RFR), Adaboost (Ada), Stacking, MLP and CNN.

GBLUP The GBLUP method was built by the following equation [26]:

1 $${{\mathbf{y}} = {\mathbf{1}}{\upmu } + {\mathbf{Za}} + {\mathbf{e}},}$$

where **1** denotes the vector of ones; $\upmu$ signifies the overall mean; **Z** is the incidence matrix that links **a** to **y**; $\mathbf{a}\sim \text{N}(\bf{0}, {\mathbf{G}\upsigma }_{\text{g}}^{2})$ is the random additive genetic effects, where **G** is the relationship matrix based on SNP markers and ${\upsigma }_{\text{g}}^{2}$ is genetic variance, $\mathbf{G}=\frac{{\bf\text{X}}^{\text{T}}\bf\text{X}}{2\sum {\text{p}}_{\text{i}}(1-{\text{p}}_{\text{i}})}$, where **X** is the centered genotype matrix; and ${\text{p}}_{\text{i}}$ signifies frequency of i-th SNP; $\mathbf{e}\sim \text{N}(\bf{0},{\mathbf{I}\upsigma }_{\text{e}}^{2})$ is the vector of random errors, where **I** is an identity matrix and ${\upsigma }_{\text{e}}^{2}$ is the residual variance. The solution for GBLUP can be written as

2 $$\begin{array}{c}\widehat{\mathbf{a}}={\left({\mathbf{Z}}^{\text{T}}{\mathbf{R}}^{-1}\mathbf{Z}+\mathbf{G}\right)}^{-1}{\mathbf{Z}}^{\text{T}}{\mathbf{R}}^{-1}\left(\mathbf{y}-\bf{1}\widehat{\upmu }\right),\end{array}$$

where $\mathbf{R}={\mathbf{I}\upsigma }_{\text{e}}^{2}$, $\widehat{\upmu }$ is the estimated overall mean of the phenotypic values.

Kernel ridge regression (KRR) The original optimization problem of KRR is as follows [27]:

3 $$\begin{array}{c}\underset{{\varvec{\upbeta}}\in {\mathbf{R}}^{\text{p}}}{\text{min}}\frac{1}{2}\uplambda {\Vert {\varvec{\upbeta}}\Vert }_{2}^{2}+\frac{1}{2\text{m}}\sum_{\text{i}=1}^{\text{m}}{\left({\text{y}}_{\text{i}}-{\upphi \left({\mathbf{x}}_{\text{i}}\right)}^{\text{T}}{\varvec{\upbeta}}\right)}^{2},\end{array}$$

where $\uplambda>0$ is the regularization constant, $\upphi$ is a mapping that maps ${\mathbf{x}}_{\text{i}}$ to higher dimensional feature space, ${\varvec{\upbeta}}$ refers to the regression coefficients in the feature space. By taking the derivative concerning ${\varvec{\upbeta}}$ equating the resulting equations to zero, the output weight vector ${\varvec{\upbeta}}$ is obtained as:

4 $$\begin{array}{c}\widehat{{\varvec{\upbeta}}}={\left({{\varvec{\upphi}}}^{\text{T}}{\varvec{\upphi}}+\uplambda \mathbf{I}\right)}^{-1}{{\varvec{\upphi}}}^{\text{T}}\bf{\text{y}},\end{array}$$

where **I** is an identity matrix of $\text{m}\times \text{m}$ dimension,${\varvec{\upphi}}=(\upphi \left({\mathbf{x}}_{1}\right),\upphi \left({\mathbf{x}}_{2}\right),\cdots ,\upphi \left({\mathbf{x}}_{\text{m}}\right) )$, we denote ${{\varvec{\upphi}}}^{\text{T}}{\varvec{\upphi}}$ in (4) as **K**, and ${\text{K}}_{\text{ij}}=\text{K}\left({\mathbf{x}}_{\text{i}},{\mathbf{x}}_{\text{j}}\right)={\upphi ({\mathbf{x}}_{\text{i}})}^{\text{T}}\cdot\upphi ({\mathbf{x}}_{\text{i}})$,$\text{i},\text{j}=\text{1,2},\cdots ,\text{m}$. Three KRR were used in this study: KRR with rbf kernel ($\text{K}\left({\mathbf{x}}_{\text{i}},{\mathbf{x}}_{\text{j}}\right)=\text{exp}\left(-\upgamma {\Vert {\mathbf{x}}_{\text{i}}-{\mathbf{x}}_{\text{j}}\Vert }^{2}\right)$), cosine kernel (${\text{K}\left({\mathbf{x}}_{\text{i}},{\mathbf{x}}_{\text{j}}\right)=\frac{\langle {\mathbf{x}}_{\text{i}},{\mathbf{x}}_{\text{j}}\rangle }{\Vert {\mathbf{x}}_{\text{i}}\Vert \cdot \Vert {\mathbf{x}}_{\text{j}}\Vert }}$) and sigmoid kernel function ($\text{K}\left({\mathbf{x}}_{\text{i}},{\mathbf{x}}_{\text{j}}\right)=\text{tanh}(\upgamma {\mathbf{x}}_{\text{i}}^{\text{T}}{\mathbf{x}}_{\text{j}})$). They were named as KRR-rbf, KRR-cos and KRR-sig, respectively.

SVR The original optimization problem of SVR is as follows [28]:

5 $$\begin{array}{c}\underset{{\varvec{\upbeta}}\in {\text{R}}^{\text{p}},\text{b}\in \text{R}}{\text{min}}\frac{1}{2}{\Vert {\varvec{\upbeta}}\Vert }^{2}+C\sum_{\text{i}=1}^{\text{m}}{\mathcal{L}}_{\upvarepsilon }\left({\text{y}}_{\text{i}}-{\upphi \left({\mathbf{x}}_{\text{i}}\right)}^{\text{T}}{\varvec{\upbeta}}-\text{b}\right),\end{array}$$

where C > 0 is the regularization constant that controls the trade-off between prediction error and model complexity, ${\mathcal{L}}_{\upvarepsilon }(\text{z})$ is the $\upvarepsilon$-insensitive loss, $\upphi$ represents a nonlinear mapping function, and b is bias.

LASSO The optimization problem of LASSO can be defined as [29]:

6 $$\begin{array}{c}\underset{{\varvec{\upbeta}}\in {\text{R}}^{\text{p}}}{\text{min}}\frac{1}{2\text{m}}{\Vert \mathbf{y}-\mathbf{X}{\varvec{\upbeta}}\Vert }^{2}+\alpha {\Vert {\varvec{\upbeta}}\Vert }_{1},\end{array}$$

where ${\upalpha }> 0$ is a parameter, it determines the sparsity of the solution $\widehat{{\varvec{\upbeta}}}$. Note that LASSO can automatically conduct feature selection by adjusting α.

Elastic net Zou and Hastie [30] proposed the ENET method to combine the benefits of ${{\ell}}_{1}$ and ${{\ell}}_{2}$-norm regularization; that is

7 $$\begin{array}{c}\underset{{\varvec{\upbeta}}\in {\text{R}}^{\text{p}}}{\text{min}}\frac{1}{2\text{m}}{\Vert \mathbf{y}-\mathbf{X}{\varvec{\upbeta}}\Vert }^{2}+\alpha {(\upgamma \Vert {\varvec{\upbeta}}\Vert }_{1}+(1-\gamma ){\Vert {\varvec{\upbeta}}\Vert }_{2}^{2}),\end{array}$$

where the regularization parameter ${\upalpha }> 0$ determines the sparsity of the solution $\widehat{{\varvec{\upbeta}}}$, $\upgamma$ is the weight ratio between ${{\ell}}_{1}$ and ${{\ell}}_{2}$-norm regularization.

RFR Random forest is essentially a collection of decision trees, and each decision tree is slightly different from other trees. Random forest can reduce the risk of overfitting by averaging the prediction results of many decision trees [31]. The equation can be expressed as:

8 $${\widehat{\text{y}}}_{\text{i}}=\frac{1}{\text{M}} \sum\limits_{{{\text{k}} = 1}}^{{\text{M}}} {{\text{T}}_{{\text{k}}} } \left( {{\bf\text{x}}_{{\text{i}}} } \right) ,$$

where ${\widehat{\text{y}}}_{\text{i}}$ represents the predicted value, M represents the number of decision trees, ${\text{T}}_{\text{k}}\left({\bf{\text{x}}}_{\text{i}}\right)$ represents the predicted value by tree k-th. Then the optimization problem of RFR is:

9 $$\begin{array}{c}\text{min}\sum\limits_{{{\text{i}} = 1}}^{{\text{m}}}\left|{\text{y}}_{\text{i}}-{\widehat{\text{y}}}_{\text{i}}\right|.\end{array}$$

Adaboost Adaboost regression is an ensemble learning method, the algorithm constructs a strong overall regression model by combining multiple weak regression models. It first fits simple regression models to the data, and then iteratively updates the weights of data points that were poorly predicted by previous models. In the article, decision trees are utilized as weak learners. The final prediction is the weighted average of the predictions made by each weak learner [32]. The equation can be expressed as:

10 $${\widehat{\text{y}}}_{\text{i}}=\sum\limits_{{{\text{k}} = 1}}^{{\text{M}}} {\omega _{{\text{k}}} } {\text{f}}_{{\text{k}}} \left( {\bf{\text{x}}_{{\text{i}}} } \right),$$

where ${\widehat{\text{y}}}_{\text{i}}$ represents the predicted target value, M is the total number of weak learners in the ensemble, ${\upomega }_{\text{k}}$ is the weight assigned to the prediction of the k-th weak learner, and ${\text{f}}_{\text{k}}\left({\bf\text{x}}_{\text{i}}\right)$ is the prediction of the k-th weak learner for the input ${\bf\text{x}}_{\text{i}}$.

Stacking Stacking regression is an ensemble method that combines multiple regression models [16]. The stacking regression is performed in two steps: firstly, training multiple single ML models to get the predicted values of target variables and the predicted values are used as the input of the next level learners. Secondly, a meta-model is trained using the predictions generated by the first step. The meta-model learns how to combine the predictions of the base models and then produces the final prediction

11 $$\begin{array}{c}\widehat{\mathbf{y}}=\text{Meta}-\text{model}\left({\widehat{\mathbf{y}}}_{\text{base}-1}, {\widehat{\mathbf{y}}}_{\text{base}-2},{\widehat{\mathbf{y}}}_{\text{base}-3}\right).\end{array}$$

**Fig. 1:**
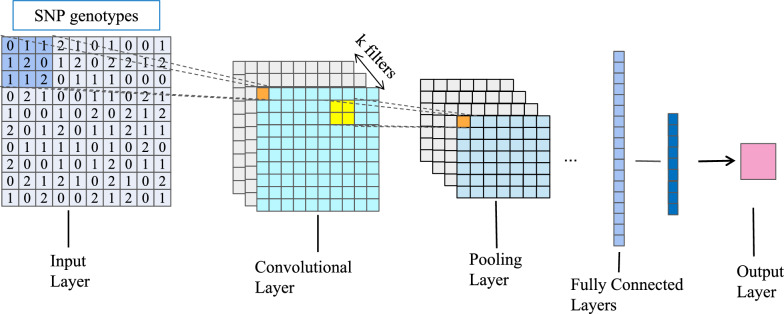
Representation of a CNN. The input layer consists of SNPs. Convolution layer consists of k filters, which capture the information in input layer by moving filters horizontally. Pooling layer involves of filters, combining the output of the previous convolution layer at certain locations into a single neuron. Fully connected layers connect every neuron in previous layer to every neuron in next layer

CNN CNN is a feedforward neural network. In CNN regression, the network consists of multiple layers, including convolutional layers, pooling layers, and fully connected layers (Fig. 1).

The network learns the optimal hyperplane by optimizing a loss function. This is done by adjusting the weights of the network through backpropagation, which computes the gradients of the loss function with respect to the weights and updates them accordingly [33].

**Fig. 2:**
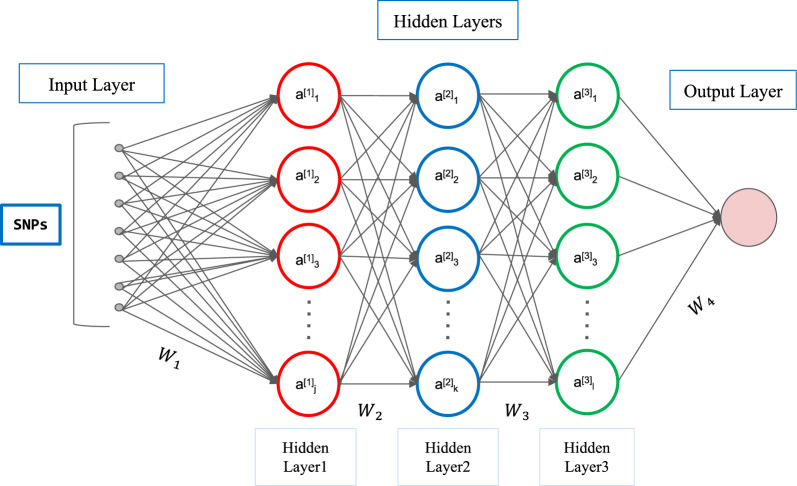
Representation of an MLP. MLP diagram with three hidden layers and a set of SNPs as input, and a basic “neuron” is illustrated with SNPs inputs. Each unit is connected to the units of previous layers by a weighted linear summation, here represented by weight matrices ${\bf\text{W}}_{\text{i}}$, and an activation function

MLP MLP composes multiple neurons into a neural network according to a certain hierarchical structure, and learns from a large amount of training data to predict the target variable (Fig. 2).

Generally, MLP regression includes input layer, hidden layer and output layer, and the number of neurons in the hidden layer and the selection of activation functions will affect the performance of the model [34].

Our experiments were conducted on the above methods, and all methods are implemented in Scikit-Learn library.

### Experimental process

**Fig. 3:**
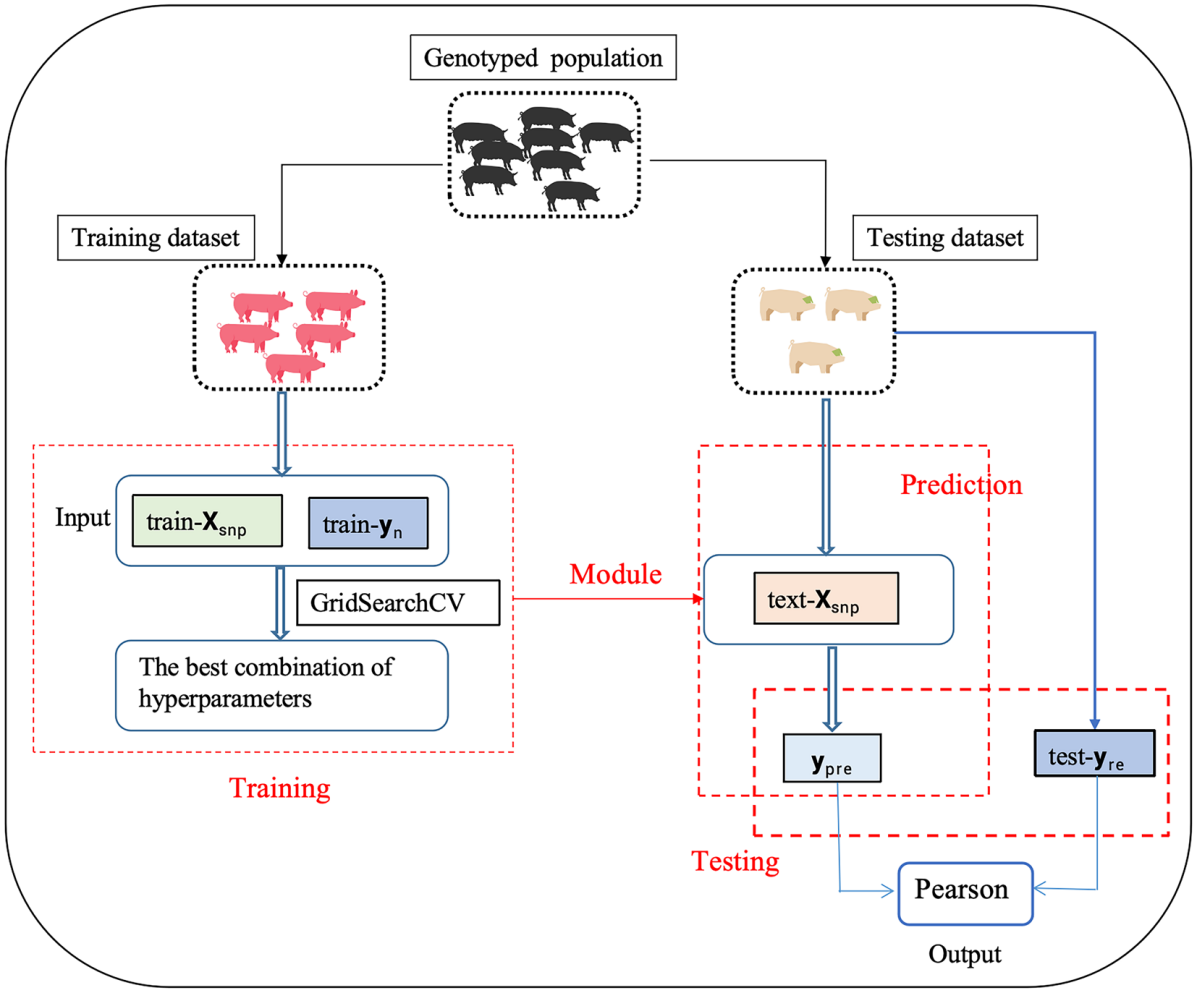
Framework for genomic prediction by machine learning algorithms. train-**X**_snp_ represents SNPs markers in the training set; train-y_n_ represents corrected phenotypes in the training set; test-**X**_snp_ represents SNPs markers in the testing set; test-**y**_re_ represents corrected phenotypes in the testing set; GEBV is genomic estimated breeding values

For all methods, randomly select 20% of D as the test set, 80% of D as the training set. During the training process, the training set was randomly divided, with 80% used for training and 20% used for validation. The basic framework is shown in Fig. 3. This process was repeated 10 times.

### Parameter setting

For ML models, the parameters C in SVR, $\uplambda$ in KRR, α in LASSO and ENET, were selected from the set: {0.001, 0.01, 0.1, 1, 10, 100, 1000}; $\upgamma$ in ENET, was selected from the set: {0.1, 0.3, 0.5, 0.7, 0.9}. The number of trees in RFR is selected from the set {100, 150, 200, 250, 300}. For Adaboost, the decision trees with maximum tree depth of 3 were used as the weak learners, the number of weak learners was select from {100, 300}, the learning rate was selected from {0.001 0.01,0.1 1}. For Stacking, KRR-rbf, KRR-cos and ridge regression (RR) were used as the weak learners, the parameters in the kernel were selected from {0.01, 0.1, 1, 10}. For all methods, on the training set, we repeated the five-fold cross-validation experiment 10 times to select the optimal parameters.

For CNN, we adopted the structure of two convolution layers followed by three fully connected layers, ReLU was used as the activation function. In the first convolution layer, the convolutional kernel is 3 × 3, stride is 2 and filters is 8. In the second convolution layer, the convolutional kernel is 3 × 3, stride is 1 and filters is 64. The number of units in the fully connected layer is 512, 256, and 1, respectively. For MLP, we adopted a structure consisting of two hidden layers followed by an output layer. ReLU activation function was applied after each hidden layer. In the first hidden layer, there are 512 units, and in the second hidden layer, there are 256 units. The output layer consists of 1 unit. For the above two DL methods, the learning rate was selected from the set {10^–5^, 10^–3^}. Additionally, the maximal number of epochs is 1000, the early stopping strategy was implemented, i.e. if the MSE on the validation set failed to decrease for 50 consecutive epochs the training process was stopped. The rationality of this strategy will be illustrated later.

### Evaluation criteria

The Pearson correlation of y^*^(real values) and **y**_pre_ (predicted values) is [34]:

12 $$\begin{array}{c}\rho \left({\mathbf{y}}^{*},{\mathbf{y}}_{\text{pre}}\right)=\frac{\text{cov}\left({\mathbf{y}}^{*},{\mathbf{y}}_{\text{pre}}\right)}{\sqrt{\text{var}\left({\mathbf{y}}^{*}\right)\text{var}\left({\mathbf{y}}_{\text{pre}}\right)}},\end{array}$$

LR statistics were used to evaluate the performance of the methods on PIC dataset which had descendant information. LR evaluates the effectiveness of a method by comparing its performance on partial and whole datasets [35]. Inspired by the literature [35, 36], the offspring individuals which constitute 20% of the PIC population composed a test set, the remaining 80% was the training set and also the whole set. We randomly selected 50% of the training samples to be the partial dataset. The statistics of LR used in this paper were, bias ${\widehat{\Delta }}_\text{p}$, slope ${\widehat{\text{b}}}_\text{p}$, correlation ${\widehat{\rho }}_{\text{w},\text{p}}$, the relative increase in accuracy resulting (RIA) ${\text{Ria}}_{\text{p}}$ [36]. The formulas of them are as follows:

13 $$\begin{array}{c}{\widehat{\Delta }}_{\text{p}}=\overline{{\widehat{\mathbf{u}} }_{\text{p}}}-\overline{{\widehat{\mathbf{u}} }_{\text{w}}} ,\end{array}$$

14 $$\begin{array}{c}{\widehat{\text{b}}}_{\text{p}}\text{=}\frac{\text{cov(}{\widehat{\text{u}}}_{\text{w, }}{\widehat{\text{u}}}_{\text{p}}\text{)}}{\text{var(}{\widehat{\text{u}}}_{\text{p}}\text{)}},\end{array}$$

15 $$\begin{array}{c}{\widehat{\rho }}_{\text{w},\text{p}}=\frac{\text{cov}\left({\widehat{\mathbf{u}}}_{\text{w}, }{\widehat{\mathbf{u}}}_{\text{p}}\right)}{\sqrt{\text{var}\left({\widehat{\mathbf{u}}}_{\text{p}}\right)\text{var}\left({\widehat{\mathbf{u}}}_{\text{w}}\right)}},\end{array}$$

16 $$\begin{array}{c}{\text{Ria}}_{\text{p}}=\frac{1}{{\widehat{\rho }}_{\text{w},\text{p}}}-1,\end{array}$$

where ${\widehat{\mathbf{u}}}_{\text{w}}$ is the estimation of the test samples based on the whole dataset, ${\widehat{\text{u}}}_{\text{p}}$ is the estimation of the test samples based on the partial dataset, $\overline{{\widehat{\mathbf{u}} }_{\text{w}}}$ is mean of ${\widehat{\mathbf{u}}}_{\text{w}}$, and $\overline{{\widehat{\mathbf{u}} }_{\text{p}}}$ is mean of ${\widehat{\mathbf{u}}}_{\text{p}}$.

## Results

For each dataset, we ranked the models based on their Pearson correlations, with the model achieving the highest Pearson correlation receiving a rank of 1, the second highest receiving a rank of 2, and so on. For PIC population, the results of LR statistics, including bias ${\widehat{\Delta }}_\text{p}$, slope ${\widehat{\text{b}}}_\text{p}$, correlation ${\widehat{\rho }}_{\text{w},\text{p}}$, RIA were presented. To determine the overall ranking of the models, we calculated the average rank for each model across all datasets. The model with the lowest average rank was considered the best overall, followed by the next highest ranks in ascending order.

### Results on T1–T5 traits

**Fig. 4:**
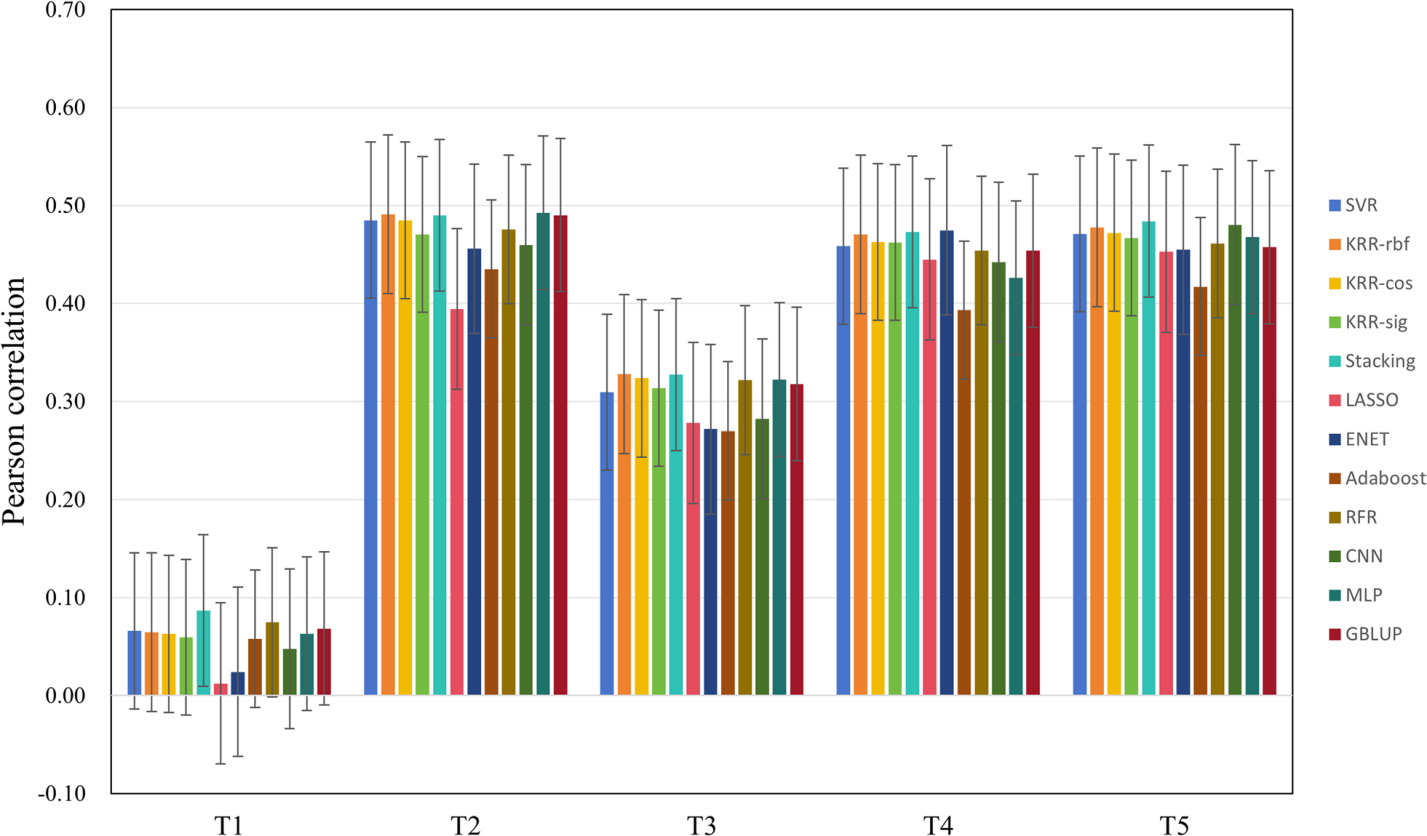
Prediction accuracy on T1-T5 traits

Figure 4 shows the Pearson correlations of 12 different methods. It is clearly that most ML methods perform better than the classical GBLUP on T1-T5 as they have higher bars in Fig. 4.

Compared with traditional GBLUP, ML methods have a maximum accuracy improvement of 3% and a minimum improvement of 0.25%. Notably, the performance of LASSO, ENET, and Adaboost on traits T1-T5 is generally inferior compared to other ML models. In Table 2, based on the average rank, the Stacking model performs the best, with an average rank of 2, followed by KRR-rbf, with an average rank of 2.8. In contrast, LASSO, Ada, and ENET perform relatively poorly, with average ranks of 10.8, 11.2, and 8.6, respectively.

**Table 2:** Rank of 12 methods on T1-T5 traits

	SVR	KRR-rbf	KRR-cos	KRR-sig	Stacking	LASSO	ENET	Ada	RFR	CNN	MLP	GBLUP
T1	4	5	7	8	1	12	11	9	2	10	6	3
T2	5	2	6	8	4	12	10	11	7	9	1	3
T3	8	1	3	7	2	10	11	12	5	9	4	6
T4	6	3	4	5	2	9	1	12	7	10	11	7
T5	5	3	4	7	1	11	10	12	8	2	6	9
Average	5.6	2.8	4.8	7	2	10.8	8.6	11.2	5.8	8	5.6	5.6

^a^Rank is based on Pearson correlation. T1–T5: trait 1- trait 5 of PIC population; KRR-rbf, KRR-cos, and KRR-sig: kernel ridge regression models with rbf, cosine, and sigmoid kernels, respectively;Average: the average rank of the method across multiple datasets

LR statistics are shown in Tables 3, 4, 5 and 6.

**Table 3:** The bias between ${\widehat{\text{u}}}_{\text{w}}$ and ${\widehat{\text{u}}}_{\text{p}}$

	T1	T2	T3	T4	T5	Average
GBLUP	0.0456	0.0393	0.1000	0.5441	5.4217	1.2302
SVR	0.0057	0.0909	0.0349	0.2068	1.7894	0.4255
KRR-rbf	0.0375	0.0877	0.0262	0.1020	2.4969	0.5501
KRR-cos	0.0315	0.0971	0.0329	0.0912	2.7566	0.6018
KRR-sig	0.0076	0.0591	0.0137	0.0175	2.2090	0.6990
Stacking	0.0285	0.0101	0.0824	0.2054	0.6016	0.1856
Adaboost	0.1131	0.0449	0.2275	0.1213	2.4182	0.5850
RFR	0.0105	0.0128	0.0162	0.0197	1.2195	0.2557
CNN	0.0321	0.0117	0.0359	0.4910	2.7947	0.6731
MLP	0.0097	0.0171	0.0414	0.0528	1.3263	0.2894

T1–T5: trait 1- trait 5 of PIC population; KRR-rbf, KRR-cos, and KRR-sig: kernel ridge regression models with rbf, cosine, and sigmoid kernels, respectively; Average: the average bias of the method across multiple datasets

**Table 4:** The slope of the regression of ${\widehat{\text{u}}}_{\text{w}}$ on ${\widehat{\text{u}}}_{\text{p}}$

	T1	T2	T3	T4	T5	Average
GBLUP	1.2617	0.7681	0.7114	0.9084	0.8628	0.9025
SVR	0.8688	0.8929	0.8218	1.1512	1.3039	1.0077
KRR-rbf	0.8494	0.9211	0.9063	1.1152	0.9132	0.9410
KRR-cos	0.8823	0.9887	0.9612	1.1892	0.9967	1.0036
KRR-sig	1.0549	1.4332	1.2435	1.5014	1.3088	1.3084
Stacking	0.2163	0.7583	0.6077	1.0568	0.7891	0.6856
Adaboost	0.2576	0.3894	0.3051	0.3938	0.1730	0.3038
RFR	0.4544	0.5920	0.2592	0.6306	0.3348	0.4542
CNN	1.0203	0.9435	0.9052	0.0783	0.8874	0.7669
MLP	0.0013	0.0179	0.0052	0.0024	0.0081	0.0070

T1–T5: trait 1- trait 5 of PIC population; KRR-rbf, KRR-cos, and KRR-sig: kernel ridge regression models with rbf, cosine, and sigmoid kernels, respectively; Average: the average slope of the method across multiple datasets

**Table 5:** The correlation between ${\widehat{\text{u}}}_{\text{w}}$ and ${\widehat{\text{u}}}_{\text{p}}$

	T1	T2	T3	T4	T5	Average
GBLUP	0.4502	0.6582	0.5111	0.6629	0.5072	0.5579
SVR	0.7635	0.8059	0.7487	0.8968	0.8848	0.8200
KRR-rbf	0.7812	0.8389	0.7604	0.9060	0.7905	0.8154
KRR-cos	0.7734	0.8777	0.7830	0.9151	0.8140	0.8326
KRR-sig	0.7944	0.9723	0.8970	0.9668	0.8932	0.9048
Stacking	0.1680	0.7693	0.6514	0.8811	0.7143	0.6368
Adaboost	0.2744	0.3941	0.2182	0.3367	0.2198	0.2887
RFR	0.3591	0.6039	0.2627	0.6500	0.3364	0.4424
CNN	0.9696	0.8829	0.8579	0.2081	0.8828	0.7603
MLP	0.0018	-0.0112	0.0054	-0.0020	0.0078	0.0004

T1–T5: trait 1- trait 5 of PIC population; KRR-rbf, KRR-cos, and KRR-sig: kernel ridge regression models with rbf, cosine, and sigmoid kernels, respectively; Average: the average correlation of the method across multiple datasets

**Table 6:** The RIA from partial dataset to whole dataset

	T1	T2	T3	T4	T5	Average
GBLUP	1.2287	0.5193	0.9568	0.5087	0.9716	0.8370
SVR	0.3097	0.2408	0.3356	0.1150	0.1302	0.2263
KRR-rbf	0.2802	0.1920	0.3152	0.1037	0.2650	0.2312
KRR-cos	0.2931	0.1393	0.2773	0.0928	0.2285	0.2062
KRR-sig	0.2588	0.0285	0.1149	0.0343	0.1195	0.1112
Stacking	3.3827	0.2999	0.5351	0.1352	0.4003	0.9506
Adaboost	2.6835	1.5375	3.5958	1.9714	3.5509	2.6678
RFR	1.8337	0.6559	2.8065	0.5384	1.9727	1.5614
CNN	0.0313	0.1327	0.1657	3.8065	0.1328	0.8538
MLP	11.8485	− 17.9096	28.2434	− 18.6819	25.8315	5.8664

T1–T5: trait 1- trait 5 of PIC population; KRR-rbf, KRR-cos, and KRR-sig: kernel ridge regression models with rbf, cosine, and sigmoid kernels, respectively; Average: the average correlation of the method across multiple datasets

From Table 3, it can be seen that the top four methods with the smallest average bias are Stacking, RFR, MLP and SVR. This indicates their minor sensitivity to training set scale, the robustness and good generalization capability. Conversely, GBLUP has the largest biases of 1.2302 that means GBLUP is more affected by the scale of the training set.

Table 4 shows that SVR, KRR-cos and KRR-rbf have average slope values of 1.0077, 1.0036 and 0.9410, respectively, which are closest to the expected value of 1. This indicates that these methods have correct dispersion in predicting phenotypes.

Table 5 depicts the correlation coefficients between ${\widehat{\text{u}}}_{\text{w}}$ and ${\widehat{\text{u}}}_{\text{p}}$. It is obvious that the KRR with different kernels and SVR have a relatively high correlation. This indicates strong prediction consistency of these four methods on both partial and whole datasets.

From Table 6, we can see that the RIA of KRR-sig is 11.12%, KRR-cos is 20.62% and SVR is 22.63%. So, these three methods are less affected by the size of the training set. MLP has the highest RIA which means that MLP is the most sensitive to the size of the training set.

### Results on growth traits

**Fig. 5:**
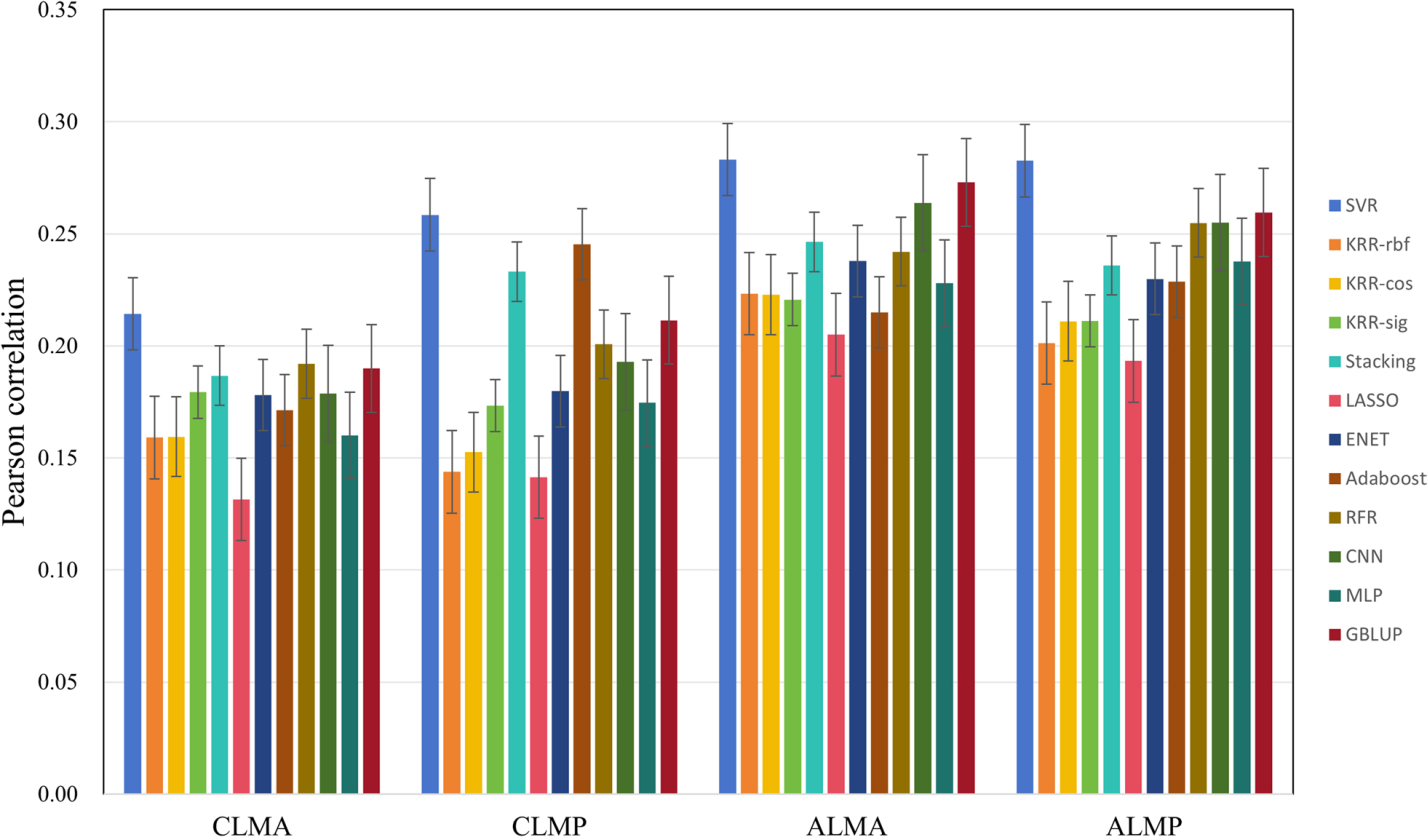
Prediction accuracy on LMA and LMP traits

**Fig. 6:**
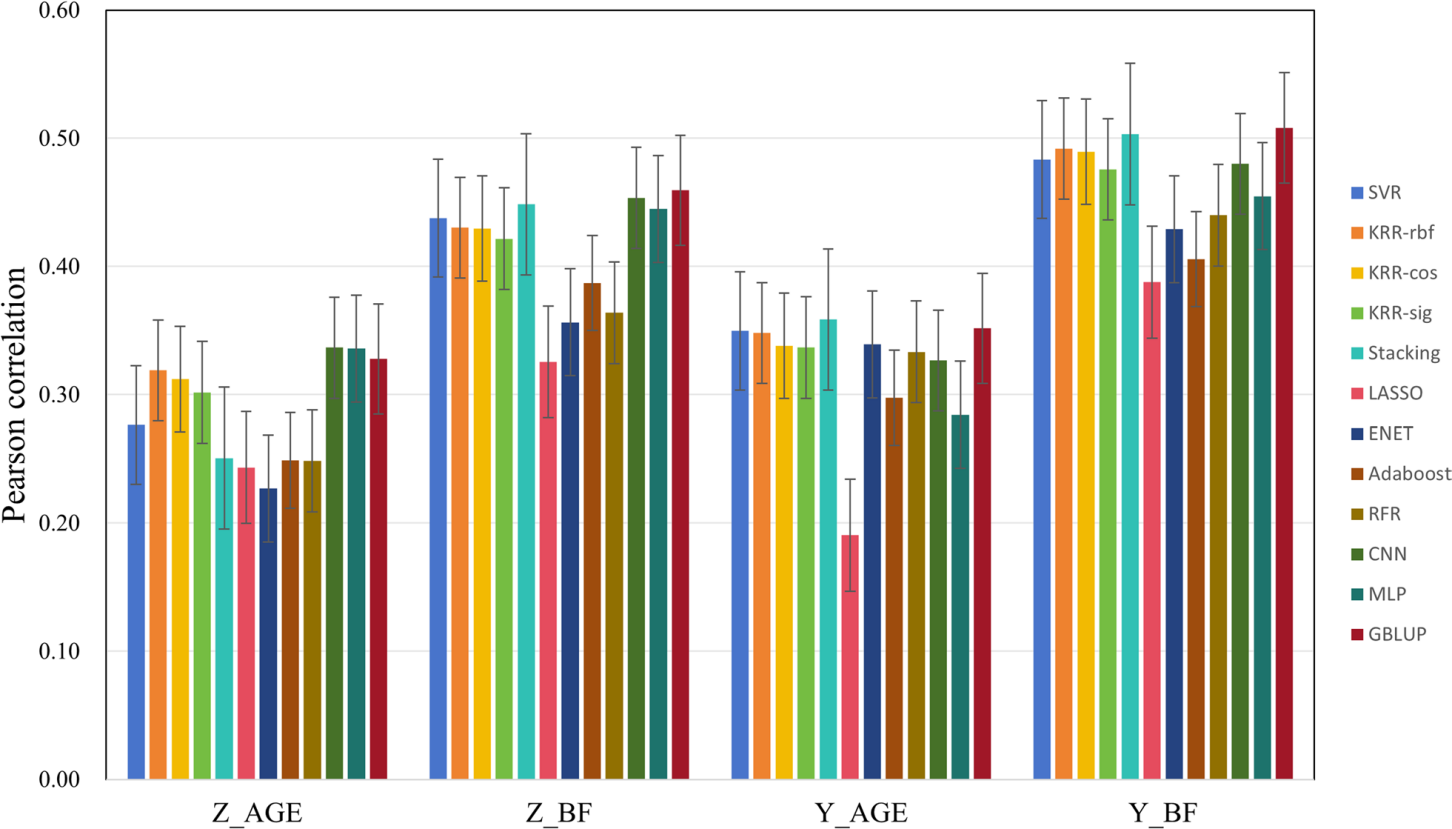
Prediction accuracy on AGE and BF traits

Figures 5 and 6 show the performance of different methods on the growth traits. We can see that some ML methods have comparable effects to GBLUP, such as KRR with different kernels and SVR. GBLUP performs the best with an average rank of 2.25 (Table 7), followed by SVR with an average rank of 3.00. Stacking and CNN also perform well, with average ranks of 3.88 and 4.50, respectively.

**Table 7:** Rank of 12 methods on all growth traits

	SVR	KRR-rbf	KRR-cos	KRR-sig	Stacking	LASSO	ENET	Ada	RFR	CNN	MLP	GBLUP
CLMA	1	11	10	5	4	12	7	8	2	6	9	3
CLMP	1	11	10	9	3	12	7	2	5	6	8	4
ALMA	1	8	9	10	4	12	6	11	5	3	7	2
ALMP	1	11	10	9	6	12	7	8	4	3	5	2
Z_AGE	7	4	5	6	8	11	12	9	10	1	2	3
Z_BF	5	6	7	8	3	12	11	9	10	2	4	1
Y_AGE	3	4	6	7	1	12	5	10	8	9	11	2
Y_BF	5	3	4	7	2	12	10	11	9	6	8	1
Average	3.00	7.25	7.63	7.63	3.88	11.88	8.13	8.50	6.63	4.50	6.75	2.25

^a^Rank is based on Pearson correlation. Z_AGE and Z_BF: 100 kg age and backfat of Large white population 1; Y_AGE and Y_BF: 100 kg age and backfat of Large white population 2; ALMA and ALMP: loin muscle area and loin muscle depth of American origin pigs; CLMA and CLMP: loin muscle area and loin muscle depth of Canadian origin pigs

KRR-rbf, KRR-cos, and KRR-sig: kernel ridge regression models with rbf, cosine, and sigmoid kernels, respectively;

Average: the average rank of the method across multiple datasets; Ada: Adaboost

### Results on reproductive traits

**Fig. 7:**
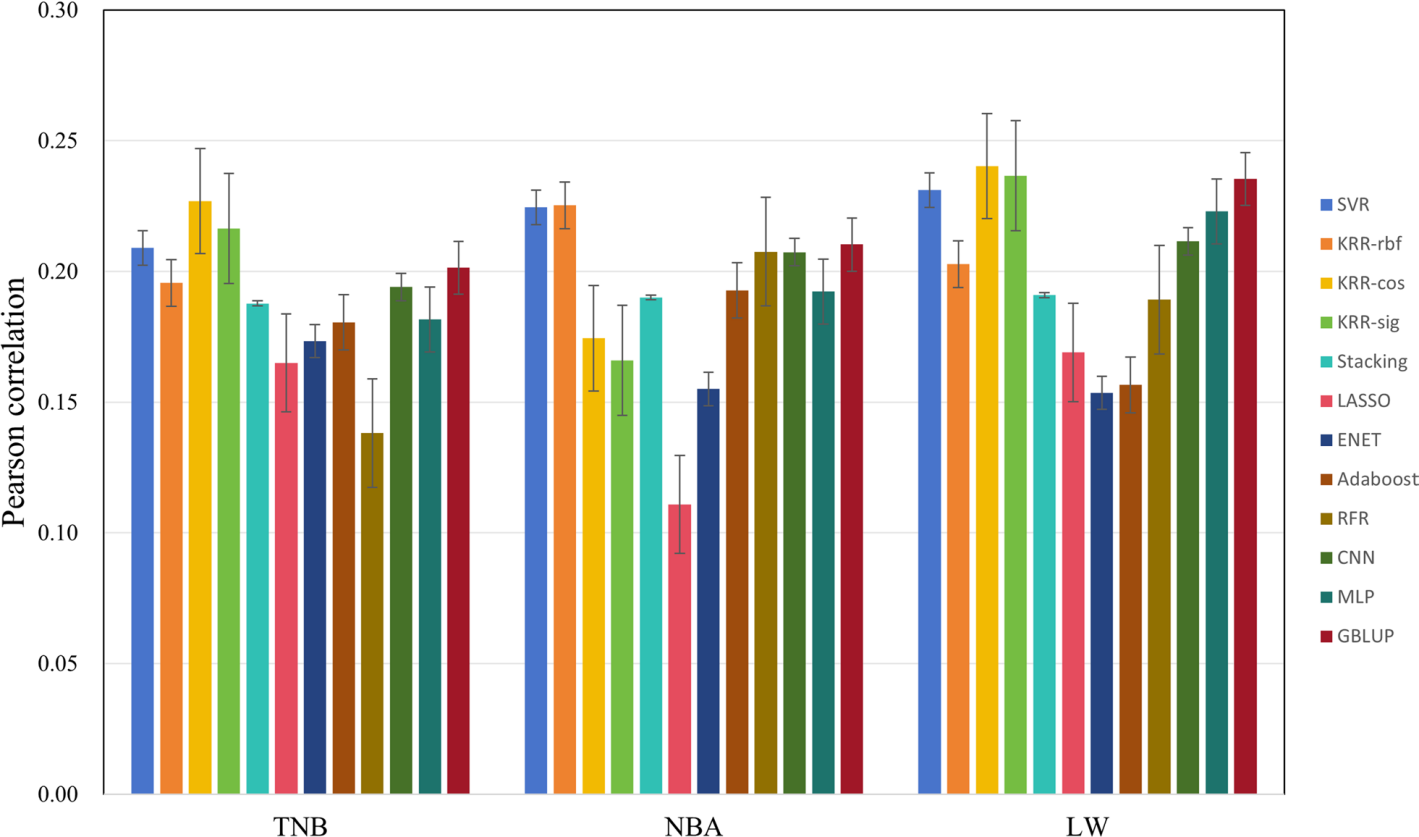
Prediction accuracy on reproductive traits

In Fig. 7, the top three methods with the high correlations on TNB are KRR-cos, KRR-sig and SVR, the average correlation of these three methods has an improvement of 1.6% compared to GBLUP.

On NBA and LW traits, KRR and SVR perform better than GBLUP. SVR performs the best with an average rank of 3.00 (Table 8), closely followed by KRR-cos with an average rank of 3.67, and GBLUP with an average rank of 3.33.

**Table 8:** Rank of 12 methods on reproductive traits

	SVR	KRR-rbf	KRR-cos	KRR-sig	Stacking	LASSO	ENET	Ada	RFR	CNN	MLP	GBLUP
TNB	3	5	1	2	7	11	10	9	12	6	8	4
NBA	2	1	9	10	8	12	11	6	4	5	7	3
LW	4	7	1	2	8	10	12	11	9	6	5	3
Average	3.00	4.33	3.67	4.67	7.67	11.00	11.00	8.67	8.33	5.67	6.67	3.33

^a^Rank is based on Pearson correlation. KRR-rbf, KRR-cos, and KRR-sig: kernel ridge regression models with rbf, cosine, and sigmoid kernels, respectively; TNB: the total number of born piglets; NBA: number of born alive piglets; LW: liter weight at birth; Average: the average rank of the method across multiple datasets; Ada: Adaboost

## Discussion

This study aimed to predict phenotypic values using a variety of ML algorithms. These ML algorithms encompass both linear and nonlinear models, as well as single algorithms and ensembled algorithms. Additionally, two types of DL algorithms (CNN and MLP) with distinct architectures were also employed. In our study, two cross validation methods were adopted, one was random cross validation and the other considered using previous generation to predict future generations in LR test. The training and test sets were defined by randomly splitting across family rather than within family. If the validation and test sets are split within families, the model may exploit the shared characteristics within these families, leading to an inflated predictive accuracy. However, this accuracy does not accurately reflect the model's generalization ability to unseen families, potentially leading to suboptimal performance in real-world applications. For T1-T5 traits (Table 2), the Stacking model exhibits the highest predictive accuracy, followed closely by KRR-rbf. Both Stacking and KRR-rbf outperform GBLUP. According to the LR statistical analysis results (Tables 3, 4, 5 and 6), the SVR, Stacking and KRR methods are more stable and less affected by the data size. This is also consistent with previous studies [15, 16, 37]. Based on the average rank of all traits (Table 9), SVR has the best rank among ML methods. Above results are consistent with previous studies [7, 15, 38]. For growth traits, GBLUP has the highest average rank, followed closely by SVR and Stacking. Notably, for the American and Canadian origin Duroc populations, SVR achieves the best performance. For reproductive traits, SVR has the highest average rank, and GBLUP, KRR-cos, and KRR-rbf follow it closely. Specially, KRR-cos shows superior prediction accuracy on TNB and LW traits; KRR-rbf performs better for NBA, suggesting that KRR is also a better method for reproductive trait prediction.

**Table 9:** Rank of 12 methods on all traits

	SVR	KRR-rbf	KRR-cos	KRR-sig	Stacking	LASSO	ENET	Ada	RFR	CNN	MLP	GBLUP
Average	3.81	5.31	6.00	6.88	4.00	11.38	8.81	9.38	6.69	5.81	6.38	3.50

^a^Rank is based on Pearson correlation. KRR-rbf, KRR-cos, and KRR-sig: kernel ridge regression models with rbf, cosine, and sigmoid kernels, respectively; Average: the average rank of the method across multiple datasets; Ada: Adaboost

We find that all ML methods with better performance in the numerical experiments involve kernel trick. Our findings are consistent with some literatures. An et al. [20] developed a cosine kernel–based KRR (KCRR), and demonstrated that KCRR performed well in both prediction accuracies and computational efficiency across different traits and multiple species. Wang et al. [10] used SVR and KRR as weak learners of Adaboost.R, found that for NBA trait, Adaboost.R2_KRR performed significantly better than ssGBLUP. Even among ML methods, Adaboost.R2_KRR consistently performed well. Additionally, the superiority of the rbf kernel model over the linear kernel lies in its ability to capture smaller, more complex marker main effects and marker-specific interaction effects [17].

Interestingly, LASSO, ENET and GBLUP are all linear models, but the effects of LASSO and ENET are not as good as GBLUP. The main reasons may be that the feature selection mechanisms of LASSO and ENET exclude features with smaller contributions during the solving process [39]. However, the excluded features may still contain valuable genetic information, which have genetic effects that influence prediction accuracy. In contrast, GBLUP is a method based on mixed linear models, which can more comprehensively utilize all feature information in the data.

**Fig. 8:**
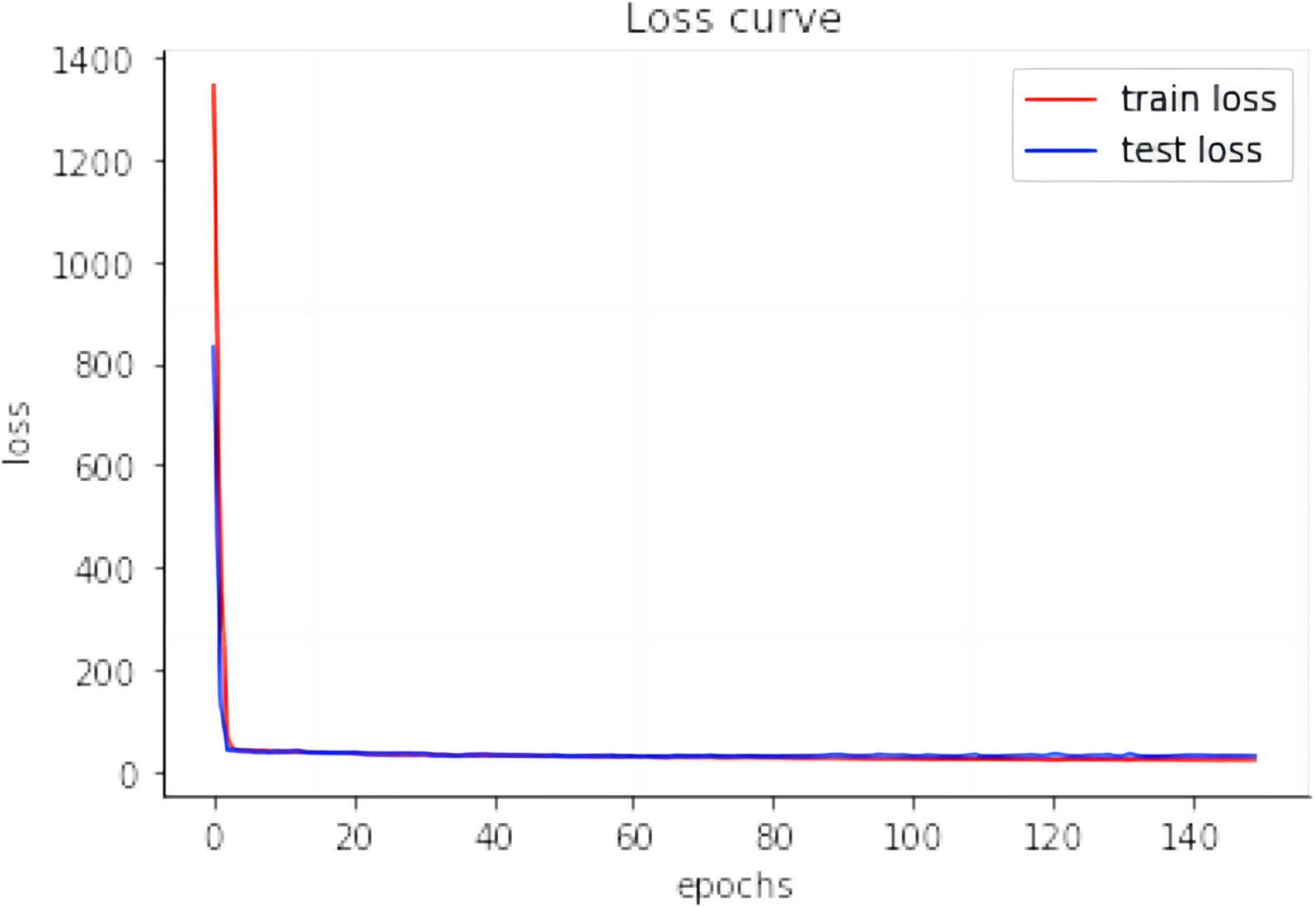
Loss curves of CNN on ALMA trait

**Fig. 9:**
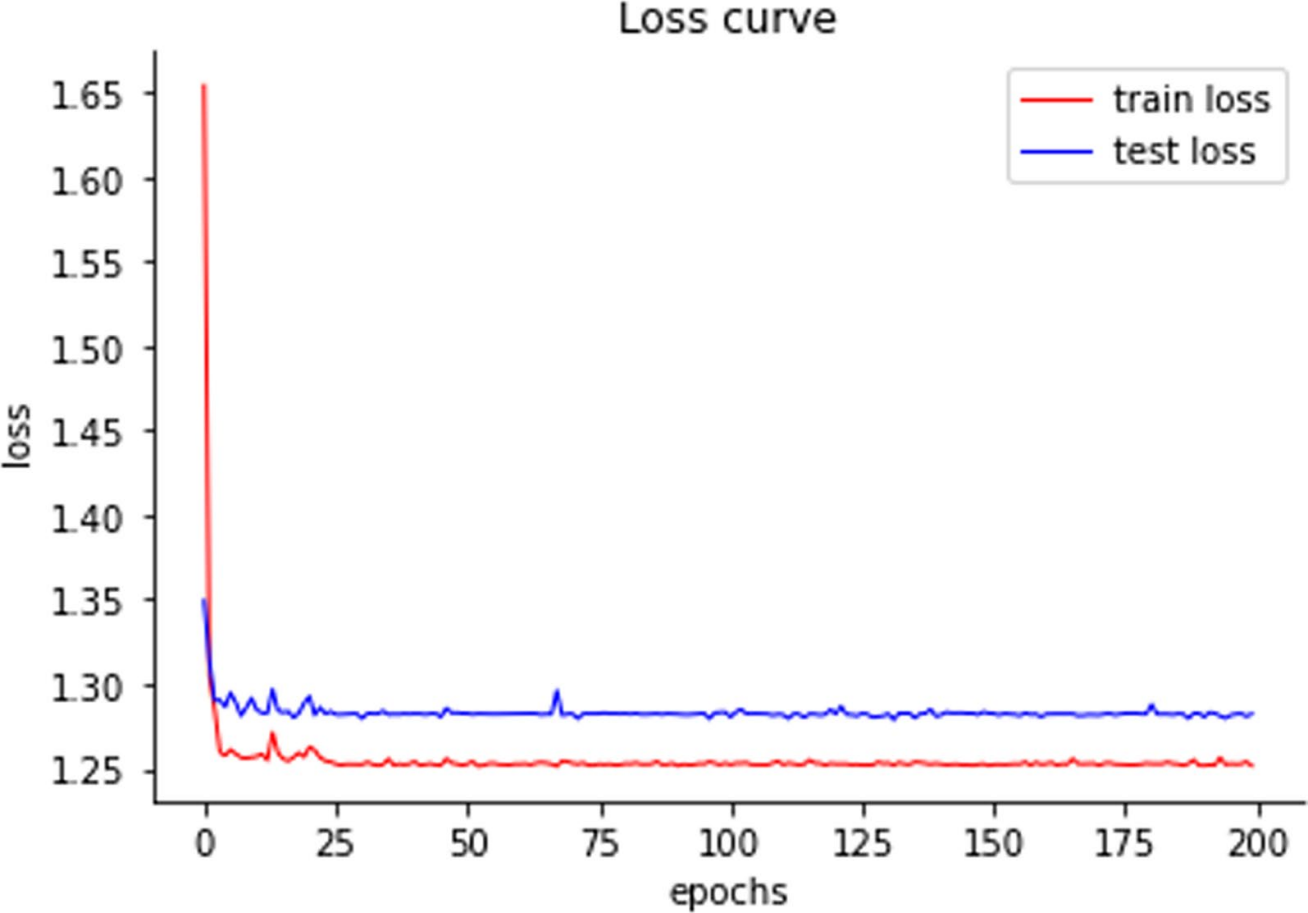
Loss curves of MLP on T2 trait

For DL methods (CNN and MLP), both training and validation losses were computed at the end of each epoch. Early stopping strategy entailed monitoring performance metrics on the validation set and halting training if the MSE on the validation set failed to decrease for 50 consecutive epochs. This approach ensured that the model did not overfit the training data and maintained its generalization capability [40]. When CNN model was used on ALMA trait and MLP was used on T2 trait, the loss curves were plotted for both the training and test sets (Figs. 8 and 9).

## Limitations

In this study, when using reproductive trait data, we restricted three reproductive traits to the data of the first litter size to ensure direct comparison between different models. However, we acknowledge that this approach may have certain implications for the generalizability of the results. Limiting the analysis to specific features may result in limited applicability of our predictions in other scenarios. To ensure a fair comparison of algorithm performance, we analyzed reproductive traits separately from other traits.

Additionally, tuning hyperparameters is crucial for ML methods. With changes in the dataset, optimal hyperparameters often require adjustment. This is particularly true for MLP and CNN algorithms, where the selection and adjustment of hyperparameters significantly impact performance. Automating the process of hyperparameter optimization during ML operations can greatly enhance efficiency and broaden the application of ML methods in genomic prediction [9].

## Conclusions

In this paper, we evaluated the performances of eleven ML algorithms and GBLUP on different traits in pigs. The results show that GBLUP and ML methods each have their own advantages. GBLUP ranks highest on two traits, SVR ranks highest on four growth traits, Stacking ranks highest on three traits, and KRR ranks highest on two traits. In addition, LR statistical analysis show that Stacking, SVR and KRR are stable. Therefore, when applying ML methods for phenotypic values prediction in pigs, we recommend these three approaches. We expect that this study could provide valuable reference for researchers in the pig breeding field when selecting ML algorithms for phenotypic values prediction.

## Data Availability

PIC pigs: 10.1534/g3.111.001453. Large White Population 1: 10.1093/g3journal/jkac118. Large White Population 2: 10.1111/age.13131. Duroc lines: 10.6084/m9.figshare.8019551.v1.

## References

[1] Meuwissen T. Genomic selection: marker assisted selection on a genome wide scale. J Anim Breed Genet. 2007;124:321–2. 18076468 10.1111/j.1439-0388.2007.00708.x

[2] Meuwissen TH, Hayes BJ, Goddard M. Prediction of total genetic value using genome-wide dense marker maps. Genetics. 2001;157:1819–29. 11290733 10.1093/genetics/157.4.1819 PMC1461589

[3] Hayes BJ, Bowman PJ, Chamberlain AJ, Goddard ME. Genomic selection in dairy cattle: progress and challenges. J Dairy Sci. 2009;92:433–43. 19164653 10.3168/jds.2008-1646

[4] Christensen OF, Lund MS. Genomic prediction when some animals are not genotyped. Genet Sel Evol. 2010;42:2. 20105297 10.1186/1297-9686-42-2 PMC2834608

[5] Zhu B, Zhu M, Jiang J, Niu H, Wang Y, Wu Y, et al. The impact of variable degrees of freedom and scale parameters in Bayesian methods for genomic prediction in Chinese Simmental beef cattle. PLoS ONE. 2016;11: e0154118. 27139889 10.1371/journal.pone.0154118 PMC4854473

[6] Gianola D, Okut H, Weigel KA, Rosa GJ. Predicting complex quantitative traits with Bayesian neural networks: a case study with Jersey cows and wheat. BMC Genet. 2011;12:87. 21981731 10.1186/1471-2156-12-87 PMC3474182

[7] Bayer PE, Petereit J, Danilevicz MF, Anderson R, Batley J, Edwards D. The application of pangenomics and machine learning in genomic selection in plants. Plant Genome. 2021;14: e20112. 34288550 10.1002/tpg2.20112 PMC12807306

[8] Zhao W, Lai X, Liu D, Zhang Z, Ma P, Wang Q, et al. Applications of support vector machine in genomic prediction in pig and maize populations. Front Genet. 2020;11: 598318. 33343636 10.3389/fgene.2020.598318 PMC7744740

[9] Lee W, Ham Y, Ban T-W, Jo O. Analysis of growth performance in swine based on machine learning. IEEE Access. 2019;7:161716–24.

[10] Wang X, Shi S, Wang G, Luo W, Wei X, Qiu A, et al. Using machine learning to improve the accuracy of genomic prediction of reproduction traits in pigs. J Anim Sci Biotechnol. 2022;13:60. 35578371 10.1186/s40104-022-00708-0 PMC9112588

[11] Ruchay A, Gritsenko S, Ermolova E, Bochkarev A, Ermolov S, Guo H, et al. A comparative study of machine learning methods for predicting live weight of duroc, landrace, and yorkshire pigs. Animals (Basel). 2022;12:1152. 35565577 10.3390/ani12091152 PMC9104573

[12] González-Recio O, Forni S. Genome-wide prediction of discrete traits using Bayesian regressions and machine learning. Genest Sel Evol. 2011;43:7. 10.1186/1297-9686-43-7 PMC3400433 21329522

[13] Abdollahi-Arpanahi R, Gianola D, Peñagaricano F. Deep learning versus parametric and ensemble methods for genomic prediction of complex phenotypes. Genet Sel Evol. 2020;52:12. 32093611 10.1186/s12711-020-00531-z PMC7038529

[14] Lee HJ, Lee JH, Gondro C, Koh YJ, Lee SH. deepgblup: joint deep learning networks and gblup framework for accurate genomic prediction of complex traits in Korean native cattle. Genet Sel Evol. 2023;55:56. 37525091 10.1186/s12711-023-00825-y PMC10392020

[15] Xiang T, Li T, Li J, Li X, Wang J. Using machine learning to realize genetic site screening and genomic prediction of productive traits in pigs. FASEB J. 2023;37: e22961. 37178007 10.1096/fj.202300245R

[16] Liang M, Chang T, An B, Duan X, Du L, Wang X, et al. A stacking ensemble learning framework for genomic prediction. Front Genet. 2021;12: 600040. 33747037 10.3389/fgene.2021.600040 PMC7969712

[17] González-Camacho JM, Ornella L, Pérez-Rodríguez P, Gianola D, Dreisigacker S, Crossa J. Applications of machine learning methods to genomic selection in breeding wheat for rust resistance. Plant Genome. 2018. 10.3835/plantgenome2017.11.0104. 30025028 10.3835/plantgenome2017.11.0104 PMC12962436

[18] Cuevas J, Crossa J, Soberanis V, Pérez-Elizalde S, Pérez-Rodríguez P, Campos GDL, et al. Genomic prediction of genotype×environment interaction kernel regression models. Plant Genome. 2016. 10.3835/plantgenome2016.03.0024. 27902799 10.3835/plantgenome2016.03.0024

[19] Cuevas J, Montesinos-López O, Juliana P, Guzmán C, Pérez-Rodríguez P, González-Bucio J, et al. Deep kernel for genomic and near infrared predictions in multi-environment breeding trials. G3 (Bethesda). 2019;9:2913–24. 31289023 10.1534/g3.119.400493 PMC6723142

[20] An B, Liang M, Chang T, Duan X, Du L, Xu L, et al. Kcrr: a nonlinear machine learning with a modified genomic similarity matrix improved the genomic prediction efficiency. Brief Bioinform. 2021;22:132. 10.1093/bib/bbab132 33963831

[21] Pérez-Enciso M, Zingaretti LM. A guide on deep learning for complex trait genomic prediction. Genes. 2019;10:553. 31330861 10.3390/genes10070553 PMC6678200

[22] Cleveland MA, Hickey JM, Forni S. A common dataset for genomic analysis of livestock populations. G3 (Bethesda). 2012;2:429–35. 22540034 10.1534/g3.111.001453 PMC3337471

[23] Zhang Y, Zhuo Y, Ning C, Zhou L, Liu J. Estimate of inbreeding depression on growth and reproductive traits in a large white pig population. G3 (Bethesda). 2022;12:jkac118. 35551391 10.1093/g3journal/jkac118 PMC9258530

[24] Yang W, Wu J, Yu J, Zheng X, Kang H, Wang Z, et al. A genome-wide association study reveals additive and dominance effects on growth and fatness traits in large white pigs. Anim Genet. 2021;52:749–53. 34403536 10.1111/age.13131

[25] Zhuang Z, Li S, Ding R, Yang M, Zheng E, Yang H, et al. Meta-analysis of genome-wide association studies for loin muscle area and loin muscle depth in two Duroc pig populations. PLoS ONE. 2019;14: e218263. 10.1371/journal.pone.0218263 PMC6561594 31188900

[26] VanRaden PM. Efficient methods to compute genomic predictions. J Dairy Sci. 2008;91:4414–23. 18946147 10.3168/jds.2007-0980

[27] Exterkate P, Groenen PJ, Heij C, van Dijk D. Nonlinear forecasting with many predictors using kernel ridge regression. Int J Forecast. 2016;32:736–53.

[28] Thomas S, Pillai GN, Pal K. Prediction of peak ground acceleration using ϵ-SVR, ν-SVR and Ls-SVR algorithm. Geomat Nat Hazards Risk. 2017;8:177–93.

[29] Tibshirani R. Regression shrinkage and selection via the LASSO. J R Stat Soc Ser B Stat Methodol. 1996;58:267–88.

[30] Zou H, Hastie T. Regularization and variable selection via the elastic ne. J R Stat Soc Series B Stat Methodol. 2005;67:301–20.

[31] Breiman L. Random forests. Mach Learning. 2001;45:5–32.

[32] Yang Y, Gao L, Abbas M, Elkamchouchi DH, Alkhalifah T, Alturise F, et al. Innovative composite machine learning approach for biodiesel production in public vehicles. Adv Eng Softw. 2023;184: 103501.

[33] Patterson J, Gibson A. Deep learning: a practitioner’s approach. Sebastopol: O’Reilly Media, Inc; 2017.

[34] Goodfellow I, Bengio Y, Courville A. Deep learning. Cambridge: MIT press; 2016.

[35] Legarra A, Reverter A. Semi-parametric estimates of population accuracy and bias of predictions of breeding values and future phenotypes using the s method. Genet Sel Evol. 2018;50:53. 30400768 10.1186/s12711-018-0426-6 PMC6219059

[36] Bermann M, Legarra A, Hollifield MK, Masuda Y, Lourenco D, Misztal I. Validation of single-step GBLUP genomic predictions from threshold models using the linear regression method: an application in chicken mortality. J Anim Breed Genet. 2021;138:4–13. 32985749 10.1111/jbg.12507 PMC7756448

[37] Gianola D, Fernando RL, Stella A. Genomic-assisted prediction of genetic value with semiparametric procedures. Genetics. 2006;173:1761–76. 16648593 10.1534/genetics.105.049510 PMC1526664

[38] VanRaden PM, Van Tassell CP, Wiggans GR, Sonstegard TS, Schnabel RD, Taylor J, et al. Invited review: reliability of genomic predictions for North American Holstein bulls. J Dairy sci. 2009;92:16–24. 19109259 10.3168/jds.2008-1514

[39] Ranstam J, Cook JA. LASSO regression. Br J Surg. 2018;105:1348.

[40] Dodge J, Ilharco G, Schwartz R, Farhadi A, Hajishirzi H, Smith N. Fine-tuning pretrained language models: Weight initializations, data orders, and early stopping. preprint arXiv. 2020; 10.48550/arXiv.2002.06305.

